# A Stable High‐Capacity Lithium‐Ion Battery Using a Biomass‐Derived Sulfur‐Carbon Cathode and Lithiated Silicon Anode

**DOI:** 10.1002/cssc.202101069

**Published:** 2021-07-16

**Authors:** Vittorio Marangon, Celia Hernández‐Rentero, Mara Olivares‐Marín, Vicente Gómez‐Serrano, Álvaro Caballero, Julián Morales, Jusef Hassoun

**Affiliations:** ^1^ Department of Chemical Pharmaceutical and Agricultural Sciences University of Ferrara Via Fossato di Mortara 17 Ferrara 44121 Italy; ^2^ Department of Química Inorgánica e Ingeniería Química Instituto de Química Fina y Nanoquímica University of Córdoba 14071 Córdoba Spain; ^3^ Department of Ingeniería Mecánica Energética y de los Materiales University of Extremadura Centro Universitario de Mérida 06800 Mérida Spain; ^4^ Department of Química Inorgánica Facultad de Ciencias University of Extremadura 06006 Badajoz Spain; ^5^ Graphene Labs Istituto Italiano di Tecnologia Via Morego 30 16163 Genova Italy; ^6^ National Interuniversity Consortium of Materials Science and Technology (INSTM) University of Ferrara Research Unit University of Ferrara Via Fossato di Mortara, 17 44121 Ferrara Italy

**Keywords:** biomass, electrode materials, energy storage, Li-ion batteries, sulfur

## Abstract

A full lithium‐ion‐sulfur cell with a remarkable cycle life was achieved by combining an environmentally sustainable biomass‐derived sulfur‐carbon cathode and a pre‐lithiated silicon oxide anode. X‐ray diffraction, Raman spectroscopy, energy dispersive spectroscopy, and thermogravimetry of the cathode evidenced the disordered nature of the carbon matrix in which sulfur was uniformly distributed with a weight content as high as 75 %, while scanning and transmission electron microscopy revealed the micrometric morphology of the composite. The sulfur‐carbon electrode in the lithium half‐cell exhibited a maximum capacity higher than 1200 mAh g_S_^−1^, reversible electrochemical process, limited electrode/electrolyte interphase resistance, and a rate capability up to C/2. The material showed a capacity decay of about 40 % with respect to the steady‐state value over 100 cycles, likely due to the reaction with the lithium metal of dissolved polysulfides or impurities including P detected in the carbon precursor. Therefore, the replacement of the lithium metal with a less challenging anode was suggested, and the sulfur‐carbon composite was subsequently investigated in the full lithium‐ion‐sulfur battery employing a Li‐alloying silicon oxide anode. The full‐cell revealed an initial capacity as high as 1200 mAh g_S_^−1^, a retention increased to more than 79 % for 100 galvanostatic cycles, and 56 % over 500 cycles. The data reported herein well indicated the reliability of energy storage devices with extended cycle life employing high‐energy, green, and safe electrode materials.

## Graphical abstract

**Green battery**: A new Li‐ion battery exploiting a sulfur cathode derived from biomass and a silicon oxide Li‐alloy anode delivers a rather high capacity for an extended number of discharge/charge cycles.

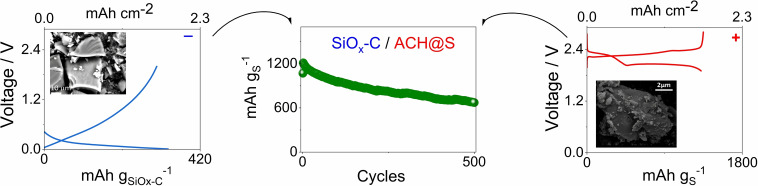

## Introduction

Lithium‐sulfur (Li−S) battery is presently considered the most promising alternative for achieving higher energy and lower cost with respect to the world‐wide employed lithium‐ion battery.^[1]^ The advantage in terms of energy density of a lithium cell using the sulfur cathode resides in the multi‐electron electrochemical conversion process [Eq. (1)], which theoretically leads to 3730 Wh kg^−1^ referring to sulfur mass.[^2^, ^3^]

(1) $$16\mathrm{Li}{}^{+}+16e{}^{-}+S{}_{8}\;\vec{\leftarrow }8\mathrm{Li}{}_{2}S$$

Instead, the typical Li‐ion cathode (e. g., layered metal oxide) (de‐)intercalates only a *x* fraction of Li^+^ ions (0<*x*<1) into the electrode structure during the electrochemical process and leads to a maximum theoretical energy density of about 900 Wh kg^−1^ (*x*=0.8) referring to the intercalated cathode mass.[^4^, ^5^] The above mentioned electrochemical process of the Li−S battery actually involves the formation of lithium polysulfides with various chain lengths (Li_2_S_*x*_, 2≤*x*≤8) as intermediates, being high‐order polysulfides able to dissolve into the electrolyte solution during cell discharge.^[6]^ During subsequent charge, these mobile species can undergo a side reduction process at the lithium surface and subsequently migrate back to the cathode, where they can be newly oxidized according to a continuous “shuttling” process leading to active material loss, electrodes degradation, decrease of delivered capacity, low coulombic efficiency, and, finally, to cell failure.[^7^, ^8^] Among the various strategies adopted to limit side reactions at the lithium metal surface including the severe shuttle process of the Li_2_S_*x*_ species, the most relevant approach has proven that the addition of LiNO_3_ as sacrificial agent to the electrolyte can protect the anode by forming a shielding solid electrolyte interphase (SEI) layer throughout a direct reduction reaction.[^9^, ^10^, ^11^, ^12^] A further very promising approach has been represented by the entrapment of sulfur in carbon matrices of various natures and morphologies to directly limit the polysulfides dissolution.[^13^, ^14^, ^15^, ^16^, ^17^] Moreover, the employment of non‐flammable and lowly‐volatile electrolyte solutions such as end‐capped glymes,[^18^, ^19^, ^20^] ionic liquids[^21^, ^22^] or polymers^[23]^ has been indicated to provide a safe and stable cycling behavior. Despite the recent notable improvements of the Li−S technology,^[24]^ the use of a lithium‐metal anode may still represent a potential safety issue that could prevent the actual use of these promising high‐energy devices. On the other hand, during the 1980s the reliability of the Li‐ion batteries has been successfully achieved by replacement of the energetic lithium‐metal anode [3860 mAh g^−1^, −3.04 V vs. standard hydrogen electrode (SHE)] with graphite to avoid the growth of metallic dendrites promoted by a heterogeneous metal deposition upon charge, possibly leading to short‐circuits and consequent cell failure.^[25]^ These researches have promoted the commercialization of the lithium‐ion battery and have been awarded in 2019 by the Nobel Prize in Chemistry.[^26^, ^27^] Thus, the application of the Li‐ion concept through the replacement of the metallic lithium with a stable and non‐reactive anode based on lithium intercalation,^[28]^ conversion,^[29]^ or alloying^[30]^ may actually represent an attractive compromise to safely exploit the multi‐electron conversion process of the Li−S battery.[^31^, ^32^, ^33^, ^34^, ^35^, ^36^] In particular, Li‐alloys with Sn[^37^, ^38^] and Si^[39]^ or their oxides[^40^, ^41^, ^42^, ^43^, ^44^] exploiting the nanostructured morphology have revealed higher capacity compared to graphite (372 mAh g^−1^), with values ranging from 500 to 1000 mAh g^−1^, due to the multiple lithium‐ion exchange per molar unit of metal. Another raising point has been represented by the sustainability of the new energy storage devices, which focused the attention on the necessity of eco‐friendly materials.[^45^, ^46^] In this respect, outstanding studies have demonstrated that carbon‐based electrodes obtained from the recycle of bio‐waste products may represent a suitable alternative to enable sustainable and, at the same time, high‐performance energy storage devices.[^47^, ^48^, ^49^] Indeed, Li−S batteries relying on cathode materials derived from crab shells,^[50]^ peanut shells,^[51]^ olive stones,^[52]^ rice husks,^[53]^ cherry pits,^[54]^ bamboo,^[55]^ brewing waste,^[56]^ and even jellyfish umbrellas have been proposed as possible alternatives.^[57]^ Taking in mind these achievements, we have explored herein the concept of a full lithium‐ion‐sulfur battery based on sustainable materials according to the most recent worldwide plans of the green economy, in particular within the UE community.^[58]^ Hence, a pre‐lithiated silicon oxide‐based anode characterized by suitable cell performances and a biomass‐derived sulfur‐carbon cathode have been coupled in a new long‐life, eco‐friendly energy storage device.[^59^, ^60^] The new sulfur‐carbon composite is initially investigated in terms of structure, morphology, thermal behavior, and applicability in lithium half‐cell. Furthermore, direct lithiation process of the silicon oxide‐based anode is exploited to allow the combination of the two electrodes, leading to an environmentally compatible lithium‐ion‐sulfur battery that may actually allow the optimization of a green and high‐performance energy storage system alternative to the conventional lithium‐ion battery.

## Results and Discussion

**Figure 1:**
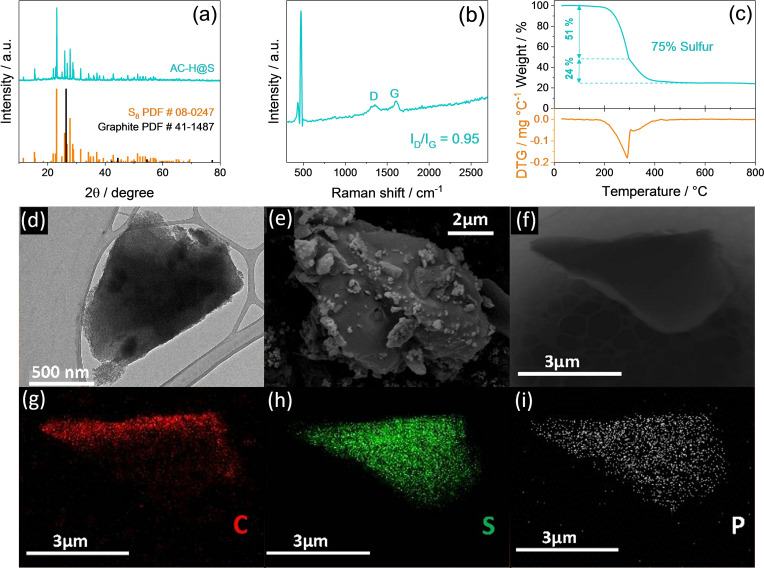
(a) XRD pattern, (b) Raman spectrum, and (c) TGA with corresponding DTG curve (bottom panel, orange left *y*‐axis) of the AC‐H@S composite powder. XRD reference data for elemental sulfur (S_8_, PDF # 8–247, orange) and graphite (PDF # 41–1487, black) are also reported for comparison. TGA was carried out under N_2_ atmosphere in the 30–800 °C temperature range at 10 °C min^−1^. (d–i) Morphological analyses of AC‐H@S powder samples: (d) TEM and (e) SEM images; (f) additional TEM image; (g–i) corresponding EDS elemental maps for (g) carbon, (h) sulfur, and (i) phosphorus.

The structural features of the sulfur‐carbon composite (AC‐H@S) are initially investigated by means of X‐ray diffraction (XRD), and the results are reported in Figure 1a. The AC‐H@S pattern exhibits the sulfur (S_8_) signals between 2*θ*=20 and 60° without any crystallographic evidence corresponding to graphite, which is usually observed at about 2*θ*=26°, as expected by the disordered nature of the activated carbon precursor (AC−H).[^60^, ^61^] Furthermore, the exclusive presence of the sulfur signals implies the absence of impurities and, thus, the effectiveness of the synthesis pathway. The disordered nature of the carbonaceous frame of AC‐H@S is confirmed by Raman spectroscopy reported in Figure 1b. Indeed, the presence of broad D (≈1350 cm^−1^) and G (≈1600 cm^−1^) bands, the related intensity ratios (*I*_D_/*I*_G_) of 0.95, as well as the absence of a defined 2D band generally observed around 2700 cm^−1^ for graphitic structures,[^62^, ^63^] indicate a large ratio of structural defects.[^60^, ^64^] The Raman spectrum also identifies the sulfur hosted in the composite, which is represented by the narrow peak centered at about 473 cm^−1^.^[65]^ The actual amount of sulfur in AC‐H@S is detected through thermogravimetric analysis (TGA) in Figure 1c, which reveals a sulfur content as high as 75 % that is expected to enable high energy density of lithium‐metal and lithium‐ion cells.^[66]^ Furthermore, the derivative thermogravimetric (DTG) curve reported in the bottom panel of Figure 1c evidences that the sulfur weight loss evolves through two subsequent steps, among which the first and major one is centered at 300 °C (51 % of the total sulfur loss) while the second one extends between 300 and 400 °C (24 % of the total sulfur loss). The first step is likely ascribable to sulfur located on the external carbon surface, whereas the second one can be related to the active material hosted within the microporous carbon structure.[^60^, ^67^, ^68^] It is worth mentioning that the above electrode architecture may actually enhance the electrical contact between the active material (i. e., sulfur) and the conductive matrix (the carbon frame), thus shortening the electron pathway and enabling the kinetics of the lithium‐sulfur electrochemical conversion process and the cell cycling.^[69]^ The electron microscopy of the AC‐H@S reported in Figure 1 shows a sample formed by submicron flakes [transmission electron microscopy (TEM) image in Figure 1d] aggregated into particles with size ranging from 1 μm or smaller to about 10 μm [scanning electron microscopy (SEM) image in Figure 1e]. Furthermore, the energy‐dispersive X‐ray spectroscopy (EDS) analyses carried out on the TEM image of Figure 1f display a uniform elemental distribution of carbon (Figure 1g) and sulfur (Figure 1h) forming the electrode matrix, as well as the above mentioned traces of phosphorous (Figure 1i) due to the H_3_PO_4_ activating agent used for the AC−H precursor synthesis.[^60^, ^70^] The observed morphology, characterized by the concomitant presence of sulfur‐carbon particles with a wide size range, may actually play an important role in achieving enhanced battery performances of the AC‐H@S composite, since the small sulfur particles generally enable high capacity values while the large ones are active material reservoirs allowing stable cycling.^[71]^

**Figure 2:**
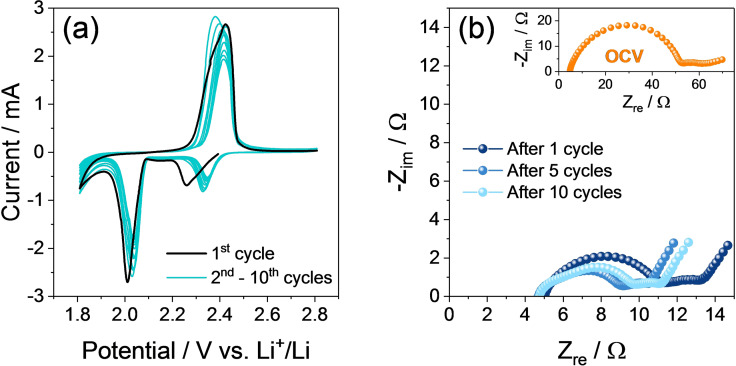
(a) CV and (b) EIS measurements performed on a Li/DOL/DME (1 : 1 *w*/*w*), 1 mol kg^−1^ LiTFSI, 1 mol kg^−1^ LiNO_3_/AC‐H@S cell. CV potential range: 1.8–2.8 V vs. Li^+^/Li; scan rate: 0.05 mV s^−1^. Impedance spectra were recorded in the 500 kHz to 100 mHz frequency range (signal amplitude: 10 mV) at OCV (inset in panel b) of the cell and after 1, 5, and 10 CV cycles.

The electrochemical features of the AC‐H@S electrode are investigated in lithium half‐cell by cyclic voltammetry (CV) and electrochemical impedance spectroscopy (EIS), as depicted in Figure 2. The first CV profile (Figure 2a) shows the typical signature of the Li−S conversion process detected by two discharge peaks at 2.25 and 2.0 V vs. Li^+^/Li, corresponding to the formation long‐chain lithium polysulfides (Li_2_S_8_ and Li_2_S_6_) and short‐chain ones (Li_2_S_*x*_, 2≤*x*≤4), respectively, reversed in a broad double‐peak between 2.3 and 2.5 V vs. Li^+^/Li during charge, which indicates the conversion of the polysulfides back to lithium and sulfur.^[72]^ The subsequent voltammetry cycles reveal a shift of the discharge peaks to higher potential values, suggesting the occurrence of an activation process that leads to a lower polarization between charge and discharge.^[69]^ The activation process is often observed taking place in the first cycles of lithium‐sulfur batteries and is usually associated with rearrangements of the sulfur electrode accompanied by a structural reorganization generally leading to the stabilization of the electrode/electrolyte interphase and the enhancement of the electrode conductivity.^[69]^ Furthermore, the narrow discharge/charge signals exhibited by AC‐H@S, as well as the notable overlapping of the potential profiles, suggest an efficient conversion process characterized by fast kinetics. Additional details on the behavior of the AC‐H@S electrode in lithium half‐cell are provided by the EIS measurements reported in Figure 2b carried out upon CV. The Nyquist plots are analyzed by non‐linear least squares (NLLS) method to obtain the corresponding equivalent circuit formed by resistive (*R*) and constant phase elements (CPE, *Q*), and identified by the *R*_e_(*R*_i_ *Q*_i_)*Q*_w_ model as reported in Table 1.[^73^, ^74^] In detail, *R*_e_ is the electrolyte resistance, identified by the high‐frequency intercept in the Nyquist plots, *R*_i_ and *Q*_i_ parallel elements (*R*_i_ *Q*_i_) represent the single or multiple high‐medium‐frequency semicircles and account for the electrode/electrolyte interphase, while *Q*_w_ indicates the Warburg‐type Li^+^ ions diffusion which is observed as a tilted line at low‐frequency values in the Nyquist plots.^[66]^ The results of NLLS analyses reported in Table 1 reveal that the above mentioned favorable activation process of the AC‐H@S electrode upon the first CV cycle is well supported by the decrease of total interphase resistance (*R*_tot_, given by the sum of the *R*_i_ elements) as well as by the corresponding Nyquist plot shrinks. Indeed, the cell exhibits a total resistance of about 40 Ω at the open‐circuit voltage (OCV; inset in Figure 2b), and a drop down to around 8 Ω after 1 cycle and 6 Ω after 10 cycles (Figure 2b). Further modifications of the electrode/electrolyte interphase upon CV may be inferred by the change of the Nyquist plot shape and the increase of the (*R*_i_ *Q*_i_) elements number in the corresponding equivalent circuit (Table 1), which is likely ascribed to a change of the electrode morphology.^[69]^

**Table 1:** NLLS analyses carried out on the EIS Nyquist plots reported in Figure 2b.^[a]^

Cell condition	Equivalent circuit	*R*_1_ [Ω]	*R*_2_ [Ω]	*R*_3_ [Ω]	*R*_tot_ ($\sum_{i=1}^{3}R$_i_) [Ω]	*χ* ^2^
OCV	*R*_e_(*R*_1_*Q*_1_)(*R*_2_*Q*_2_)*Q*_w_	32±4	8.0±3.8	–	40±8	1×10^−4^
1 CV cycle	*R*_e_(*R*_1_*Q*_1_)(*R*_2_*Q*_2_)*Q*_w_	6.1±0.1	2.3±0.1	–	8.4±0.2	6×10^−6^
5 CV cycles	*R*_e_(*R*_1_*Q*_1_)(*R*_2_*Q*_2_)(*R*_3_*Q*_3_)*Q*_w_	0.8±0.3	3.3±0.3	1.5±0.3	5.6±0.9	8×10^−5^
10 CV cycles	*R*_e_(*R*_1_*Q*_1_)(*R*_2_*Q*_2_)(*R*_3_*Q*_3_)*Q*_w_	1.0±0.1	3.8±0.2	1.3±0.1	6.1±0.4	1×10^−5^

[a] Recorded upon CV test performed on a Li/DOL/DME (1 : 1 *w*/*w*), 1 mol kg^−1^ LiTFSI, 1 mol kg^−1^ LiNO_3_/AC‐H@S cell. The analyses were carried out by applying the NLLS method through a Boukamp tool.[^73^, ^74^] The *χ* ^2^ is an adimentional number indicating the goodness of the analysis. NLLS analyses with *χ* ^2^ equal to or lower than 1×10^−4^ are acceptable.

**Figure 3:**
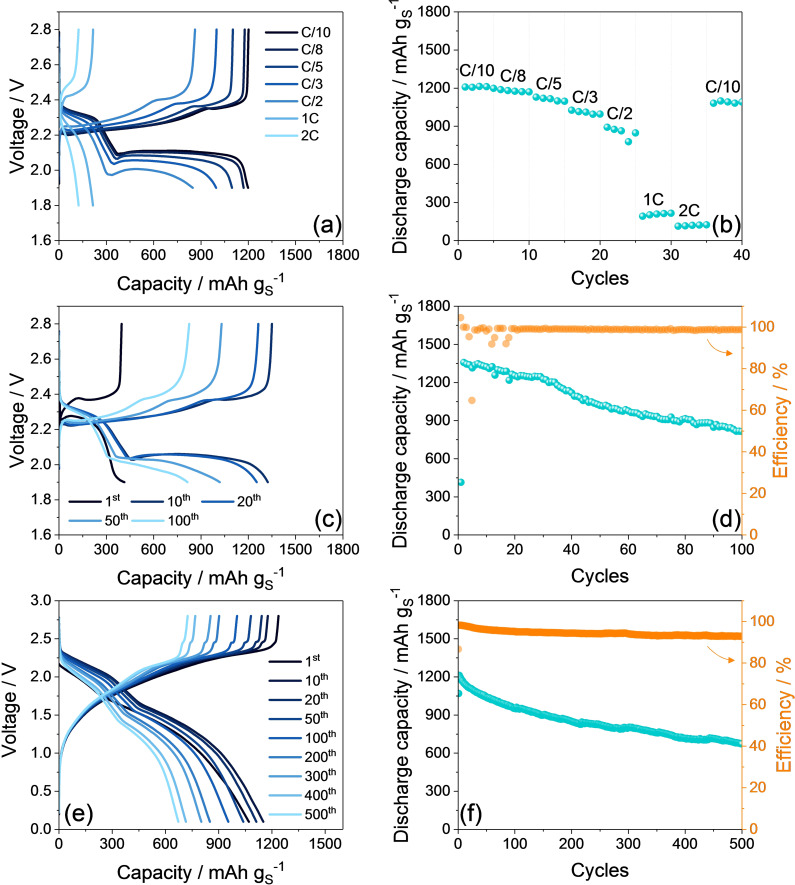
(a–d) Galvanostatic tests performed on Li/DOL/DME (1 : 1 *w*/*w*), 1 mol kg^−1^ LiTFSI, 1 mol kg^−1^ LiNO_3_/AC‐H@S half‐cells. In detail: (a,c) selected voltage profiles and (b,d) corresponding cycling trend (right *y*‐axis in panel (d) refers to coulombic efficiency) related to tests carried out (a,b) at increasing currents employing the C/10, C/8, C/5, C/3, C/2, 1 C, and 2 C rates and (c,d) at the constant current rate of C/3. Voltage ranges: 1.9–2.8 V from C/10 to C/2 and 1.8–2.8 V for 1 C and 2 C rates. (e,f) Galvanostatic tests performed on Li_*y*_SiO_*x*_−C/DOL/DME (1 : 1 *w*/*w*), 1 mol kg^−1^ LiTFSI, 1 mol kg^−1^ LiNO_3_/AC‐H@S full‐cell at a C/5 current rate. In detail: (e) selected voltage profiles and (f) corresponding cycling trend with coulombic efficiency in right *y*‐axis. Voltage range: 0.1–2.8 V. The N/P ratio between the Li_*y*_SiO_*x*_−C and AC‐H@S electrodes was tuned to a value of 1.04. The anode was electrochemically pre‐lithiated at 50 mA g^−1^ for over 30 cycles in the 0.01–2.0 V voltage range in lithium half‐cell.

The electrochemical performances of the AC‐H@S electrode in lithium half‐cell are evaluated in Figure 3 through galvanostatic tests at increasing current from C/10 (1 C=1675 mA g_S_^−1^) to C/8, C/5, C/3, C/2, 1 C, and 2 C (Figure 3a,b), and at the constant rate of C/3 for 100 cycles (Figure 3c,d). The evolution of the voltage profiles reported in Figure 3a evidences that the Li/AC‐H@S cell at a C‐rate lower than 1 C operates according to the CV of Figure 2 with two discharge plateaus centered at 2.3 and 2.0 V due to the reduction of sulfur to lithium polysulfides, and merging charge plateaus between 2.3 and 2.4 V due to the subsequent oxidation. The cell reveals the excepted increase of the discharge/charge polarization by raising the current from C/10 to C/2; however, a further increase to 1 C and 2 C turns into the deactivation of the electrochemical process due to excessive overvoltage, which is indicated by the concomitant drop of the delivered capacity. Nonetheless, the cycling trend depicted in Figure 3b shows for the AC‐H@S electrode stable capacity values of 1200, 1180, 1100, and 1000 mAh g_S_^−1^ at C/10, C/8, C/5, and C/3, respectively, and between 890 and 780 mAh g_S_^−1^ at C/2. After the abrupt decay of the delivered capacity below 300 and 150 mAh g_S_^−1^ at 1 C and 2 C, respectively, the cell recovers about 92 % of the initial value when the C‐rate is lowered back to C/10, thus suggesting a good stability of the active material by changing currents. Figure 3c shows selected voltage profiles related to the Li/AC‐H@S cell characterized at the constant current of C/3 for 100 cycles. Interestingly, the poor capacity exhibited during the first cycle (400 mAh g_S_^−1^), as well as the anomalous evolution of the corresponding discharge/charge plateaus, may be attributed to an initial low conductivity of the electrode/electrolyte interphase, which is improved by the activation process after 1 cycle as already discussed in Figure 2. On the other hand, the electrode exhibits a remarkable capacity, with starting values higher than 1200 mAh g_S_^−1^, and a coulombic efficiency approaching 99 % at the steady state (Figure 3d). However, the half‐cell shows a retention limited to 60 % upon 100 discharge/charge cycles, which may be ascribed to the reaction of the lithium metal with polysulfides or impurities such as phosphorous dissolved from the activated carbon matrix (see EDS and related discussion in Figure 1). Therefore, we may assume that the AC‐H@S electrode has suitable performance for battery application, in particular for Li‐ion cell in which the above issues ascribed to the presence and reactivity of the lithium metal can be actually mitigated. The AC‐H@S electrode is subsequently coupled with a silicon oxide‐based anode (SiO_*x*_−C) in a full lithium‐ion‐sulfur battery. Prior to using, the SiO_*x*_−C electrode was activated by galvanostatic cycling in lithium cell (see the Experimental Section) in order to obtain the lithiated Li_*y*_SiO_*x*_−C anode, which is a suitable Li^+^ions reservoir in the full Li‐ion‐sulfur cell.^[75]^ The voltage profiles of the galvanostatic test performed on the Li_*y*_SiO_*x*_−C/AC‐H@S full‐cell at the constant current rate of C/5 reported in Figure 3e reveal an electrochemical process centered at about 1.8 V. The discharge and charge processes evolve according to the combination between the typical voltage shapes associated to the multi‐step conversion process of the Li−S battery^[3]^ and the (de‐)alloying mechanism of the Li/SiO_*x*_−C cell.^[59]^ During the first cycle, the discharge exhibits two broad plateaus extending in voltage intervals of 1.9–2.3 and 1.1–1.6 V, which are reversed into a sloping charge profile evolving between 1.5 and 2.35 V. Interestingly, the subsequent cycles exhibit the gradual fragmentation of the charge plateau into three different processes taking place at 1.7, 2.2, and 2.45 V. This trend may be ascribed with structure rearrangements and consolidation of a stable interphase at the surface of both electrodes, as suggested by CV and EIS measurements in Figure 2 and by previous works.^[76]^ This complex process is likely reflected into an increase of delivered capacity upon the first cycle, as also evidenced by the cycling trend shown in Figure 3f. Indeed, the Li_*y*_SiO_*x*_−C/AC‐H@S cell exhibits 1070 mAh g_S_^−1^ during the first cycle that raise up to 1210 mAh g_S_^−1^ during the second one. The Li‐ion‐sulfur cell shows a relevant capacity retention, in particular if compared to the corresponding Li‐sulfur half‐cell. Hence, the corresponding plots in Figure 3f (Li‐ion cell) and Figure 3d (Li−S cell) indicate a retention over 100 cycles of about 79 % for the former and of 60 % for the latter. Furthermore, the test extended for 500 cycles in the Li‐ion cell (Figure 3f) reveals a residual capacity as high as 670 mAh g_S_^−1^ corresponding to a retention of 56 % of the maximum value, and a coulombic efficiency higher than 92 %. Therefore, considering an average operating voltage of about 1.8 V and a capacity of 670 mAh g_S_^−1^ after 500 cycles, we can estimate that the Li_*y*_SiO_*x*_−C/AC‐H@S cell can still hold upon this challenging test a theoretical specific energy density of about 1200 Wh kg_S_^−1^, which could lead to practical value of 400 Wh kg^−1^ by taking in consideration a correction factor of 1/3 that includes all the inactive components of the cell.^[77]^ This stable cycling behavior, the remarkable delivered capacity and energy, and the proper voltage evolution of the cell are herein achieved by tuning the negative‐to‐positive (N/P) ratio with a very limited anode excess, that is, 1.04 (see Figure S1 in the Supporting Information). The N/P ratio approaching the unity may in fact favor the achievement of optimized full‐cell performances as indicated in previous papers.[^4^, ^32^]

**Figure 4:**
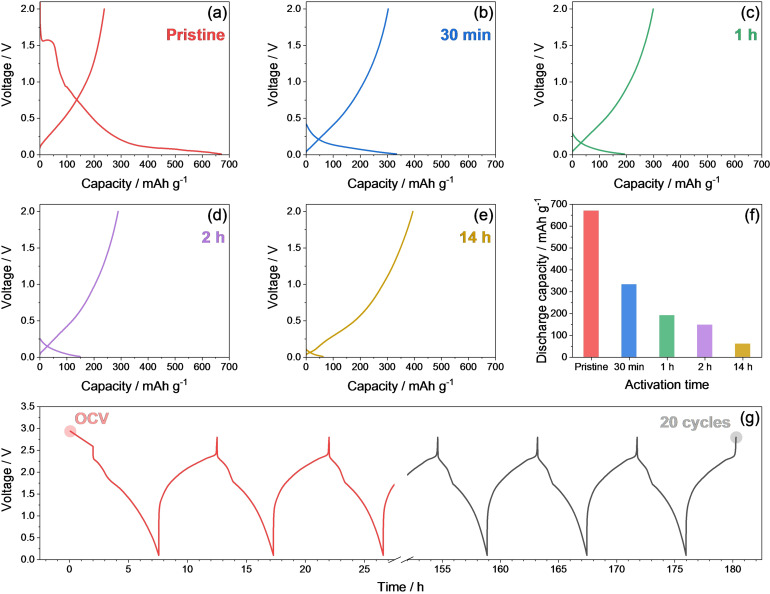
(a–f) Voltage profiles related to the first cycle of (a) a Li/DOL/DME (1 : 1 *w*/*w*), 1 mol kg^−1^ LiTFSI, 1 mol kg^−1^ LiNO_3_/SiO_*x*_−C pristine cell and (b–e) Li/DOL/DME (1 : 1 *w*/*w*), 1 mol kg^−1^ LiTFSI, 1 mol kg^−1^ LiNO_3_/Li_*y*_SiO_*x*_−C cells employing Li_*y*_SiO_*x*_−C electrodes chemically pre‐lithiated (activated) at various times: (b) 30 min, (c) 1 h, (d) 2 h, and (e) 14 h (see the Experimental Section for details). (f) Histogram representation of the discharge capacity values obtained from the cycling tests reported in panels (a–e). All the cycling measurements were carried out at a current rate of 50 mA g^−1^ (referred to the pristine SiO_*x*_−C mass) in the 0.01–2 V voltage range. (g) Voltage profiles related to the full cell exploiting the Li_*y*_SiO_*x*_−C/DOL/DME (1 : 1 *w*/*w*), 1 mol kg^−1^ LiTFSI, 1 mol kg^−1^ LiNO_3_/AC‐H@S configuration galvanostatically cycled at the constant rate of C/5 in the 0.1–2.8 V voltage range. The anode was chemically pre‐lithiated by employing an activation time of 48 h (see the Experimental Section for details).

With the aim of further understanding the behavior of the full Li‐ion‐sulfur cell, we investigate hereafter the morphological and structural features of the AC‐H@S and SiO_*x*_−C electrodes at the pristine state and after cycling. Indeed, an additional full Li_*y*_SiO_*x*_−C/AC‐H@S cell is assembled with an anode achieved by chemical pre‐lithiation of the SiO_*x*_−C material through direct contacting the electrode disk with a lithium foil soaked with the electrolyte to reach the Li_*y*_SiO_*x*_−C alloy (see the Experimental Section for further details). This activation pathway allows a rapid and efficient lithiation of the electrode,^[78]^ as demonstrated by Figure 4. The selected voltage profiles of lithium half‐cells assembled either with a pristine SiO_*x*_−C electrode (Figure 4a) or with Li_*y*_SiO_*x*_−C electrodes achieved by chemical pre‐lithiation at various time regimes, that is, 30 min (Figure 4b), and 1, 2, and 14 h (Figure 4c–e, respectively) show that the capacity delivered at the first discharge of the half‐cells, corresponding to the charge step in full‐cell, decreases by increasing the contact time, thus indicating the progressive lithiation “activation” of the SiO_*x*_−C electrode (see histogram in Figure 4f). The Li_*y*_SiO_*x*_−C/AC‐H@S full‐cell is then assembled by coupling a fresh cathode with an anode chemically activated for 48 h to ensure the complete lithiation, and galvanostatically cycled at the constant current rate of C/5 for 20 cycles (Figure 4g).

**Figure 5:**
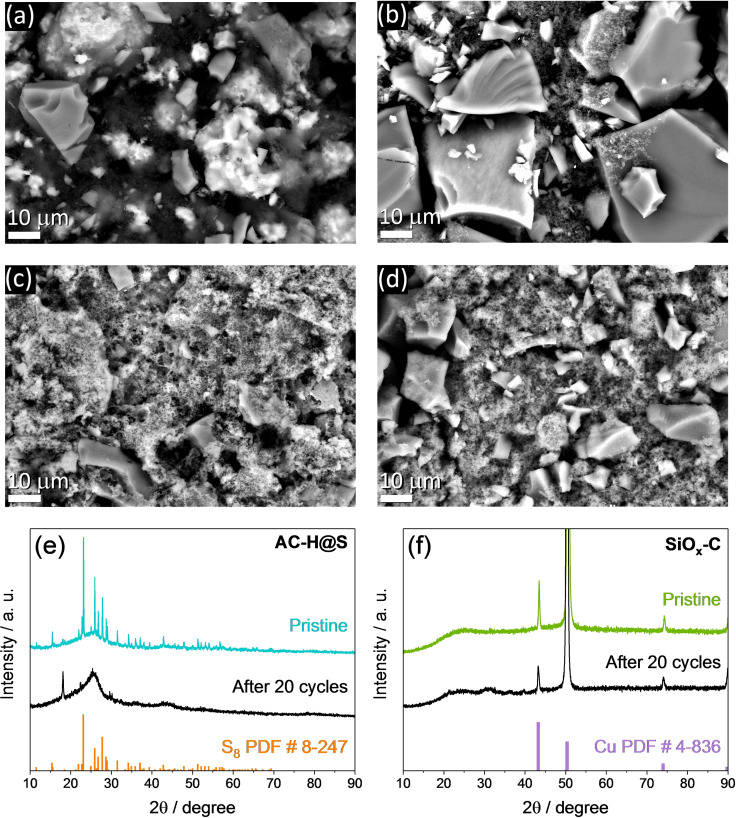
(a–d) SEM images recorded on (a,c) AC‐H@S and (b,d) SiO_*x*_−C electrodes at (a,b) the pristine state and (c,d) after 20 discharge/charge cycles in a Li_*y*_SiO_*x*_‐C/DOL/DME (1 : 1 *w*/*w*), 1 mol kg^−1^ LiTFSI, 1 mol kg^−1^ LiNO_3_/AC‐H@S cell (see the corresponding voltage profiles in Figure 4g) cycled at a constant current rate of C/5 in the 0.1–2.8 V voltage range. (e–f) XRD patterns of the (e) AC‐H@S and (f) SiO_*x*_−C electrodes before and after cycling. XRD reference data for elemental sulfur [S_8_, panel (e), PDF # 8–247] and copper [Cu, panel (f), PDF # 4–836] are also reported for comparison.

Subsequently, the two electrodes are recovered after disassembling the cycled cell and characterized along with pristine AC‐H@S and SiO_*x*_−C disks by SEM and XRD as displayed in Figure 5, while the corresponding elemental distribution is detected by EDS (Figures S2 and S3, respectively, in the Supporting Information). The SEM image related to the pristine AC‐H@S cathode (Figure 5a) reveals the presence of micrometric sulfur (bright domains) and H_3_PO_4_‐activated carbon particles (grey domains) uniformly distributed on the electrode surface, as evidenced by the EDS elemental maps of carbon (Figure S2a), sulfur (Figure S2b) and phosphorous (Figure S2c), while the one related to fluorine (Figure S2d) evidences the polyvinylidene fluoride (PVDF) added to the electrode formulation as the polymer binder (see the Experimental Section). On the other hand, the pristine SiO_*x*_−C shows large particles with size exceeding 20 μm (Figure 5b) formed by a carbon matrix containing silicon oxide particles, as identified by the EDS elemental maps of C (Figure S3a), Si (Figure S3b), and O (Figure S3c).^[59]^ Substantial modifications of the morphology can be observed in Figure 5c,d, which displays the SEM images of the AC‐H@S and SiO_*x*_−C electrodes, respectively, after cycling. Indeed, AC‐H@S shows a surface apparently filled by active material or carbon particles different than the pristine one (compare Figure 5a,c), while SiO_*x*_−C displays particles having similar shape with respect to the pristine state however with smaller size (compare Figure 5b,d), as likely associated with the unavoidable volume changes due to the Li−Si (de‐)alloying process and possible partial fragmentation. Both AC‐H@S and SiO_*x*_−C exhibit the presence of a bright uniform surface layer suggesting the growth of a SEI formed by carbon (EDS in Figures S2e and S3e), sulfur (Figure S2f and inset in Figure S3h), fluorine (Figures S2h and S3h), and oxygen (inset in Figure S2h and Figure S3g). The above elemental composition of the SEI layer can be attributed to the partial decomposition of the 1,3‐dioxolane (DOL) and 1,2‐dimethoxyethane (DME) ether chains or the lithium bis(trifluoromethanesulfonyl)imide (LiTFSI) conductive salt in the electrolyte,[^79^, ^80^] to the electrodeposition of amorphous sulfur upon charge on the AC‐H@S surface,^[66]^ as well as to possible side reaction of lithium polysulfides with the lithiated SiO_*x*_−C electrode.^[81]^ Interestingly, the XRD pattern related to the AC‐H@S electrode exhibits at the pristine state the broad peak centered at 2*θ*=26° ascribed to the porous carbon‐cloth electrode (see the Experimental Section) and the typical crystalline sulfur signals between 2*θ*=20 and 60°, which vanish in the pattern of the cycled electrode (Figure 5e). Furthermore, both the patterns related to the SiO_*x*_−C electrode (before and after cycling) show exclusive peaks ascribed to the copper support in addition to sulfur impurity detected by EDS (inset in Figure S3h), as well as the unaltered amorphous structure of the carbon‐embedded silicon oxide particles (Figure 5f). Therefore, the SEM/EDS analyses and the XRD measurements suggest specific morphological modifications and structural stability of the two electrodes upon cycling in full‐cell, and the formation of a protecting SEI layer through a series of favorable side reactions at the electrode/electrolyte interphase.

## Conclusions

A sulfur‐carbon composite indicated as AC‐H@S has been synthesized using a bio‐residues carbonaceous precursor and characterized in view of possible application in a sustainable Li‐ion‐sulfur battery. X‐ray diffraction (XRD) measurement performed on the composite powder evidenced the absence of side products and the predominant presence of sulfur, as well as the disordered nature of the carbon frame as confirmed by Raman spectroscopy. Thermogravimetric analysis of the AC‐H@S powder detected a sulfur content as high as 75 %, which is allowed by the microporous structure of the activated carbon. Cyclic voltammetry (CV) and electrochemical impedance spectroscopy (EIS) tests performed on lithium half‐cell suggested fast kinetics of the electrochemical conversion process and a remarkable conductivity of the electrode/electrolyte interphase upon activation. In particular, CV profiles identified two reversible and narrow discharge peaks at 2.25 and 2.0 V vs. Li^+^/Li reversed into a charge process extending from 2.3 to 2.5 V vs. Li^+^/Li, with interphase resistance values measured through EIS of 40 Ω at the open‐circuit voltage decreasing down to 6 Ω upon 10 CV cycles. Galvanostatic cycling tests carried out on lithium half‐cells have shown maximum capacity values exceeding 1200 mAh g_S_^−1^, coulombic efficiency approaching 99 %, and a rate capability extending up to C/2. Despite the suitability of the AC‐H@S for battery application, the half‐cell suffered by an excessive decay of the capacity by cycling due to side reactivity of the lithium metal with polysulfides and phosphorous impurities possibly dissolved into the electrolyte upon cathode operation. Therefore, the lithium electrode was replaced by a Li‐alloy anode based on silicon oxide into amorphous carbon to achieve the Li_*y*_SiO_*x*_−C/AC‐H@S cell exploiting the Li‐ion configuration. The new battery revealed at C/5 a sloped voltage signature centered at about 1.8 V in line with the combination of the multi‐step sulfur conversion and the (de‐)alloying process of the Li_*y*_SiO_*x*_−C anode. Furthermore, the Li_*y*_SiO_*x*_−C/AC‐H@S full‐cell delivered a maximum capacity of about 1200 mAh g_S_^−1^, retained slightly below 60 % over 500 cycles, with a final theoretical energy density of about 1200 Wh kg_S_^−1^ and an estimated practical value of 400 Wh kg^−1^. Such a notable performance has been herein achieved by using enhanced anode and cathode materials, and by properly tuning their negative‐to‐positive ratio to a value approaching the unity (≈1.04), thus concomitantly allowing long cycle life and high delivered capacity. Accordingly, the structural retention and the formation of a suitable solid electrolyte interphase (SEI) layer have been actually observed by performing ex‐situ XRD, scanning electron microscopy (SEM), and energy‐dispersive X‐ray spectroscopy (EDS) measurements on pristine electrodes and on materials recovered from the Li_*y*_SiO_*x*_−C/AC‐H@S cell upon cycling. Therefore, this study may represent a step forward to achieve an alternative Li‐ion battery employing environmentally friendly materials, such as sulfur, bio‐waste derivatives, and silicon, characterized by high energy and extended cycle life.

## Experimental Section

### Synthesis of the carbon precursor

The carbon precursor exploited herein was obtained by treatment of biomass residues of cherry pits provided by Asociación de Cooperativas del Valle del Jerte (Cáceres province, Spain), as reported in a previous work.^[60]^ Accordingly, the treated cherry pits powder was activated through H_3_PO_4_ treatment of the precursor,^[70]^ and the obtained sample was subsequently annealed under N_2_ and indicated as AC−H. The detailed chemical‐physical characterization of the AC−H carbon precursor is reported elsewhere.^[60]^

### Synthesis of the sulfur‐carbon composite

The sulfur‐carbon composite was obtained by infiltrating sulfur in the AC−H carbon precursor via in‐situ disproportionation of sodium thiosulfate pentahydrate (Na_2_S_2_O_3_ ⋅ 5H_2_O) in acidified aqueous solution. Accordingly, 3 g of Na_2_S_2_O_3_ ⋅ 5H_2_O (Sigma‐Aldrich) was dissolved in a solution composed of 150 mL of H_2_O and 4.5 mL of a Triton X‐100 solution (1 vol %), that is, a polymer surfactant that avoids sulfur agglomerates and allows controlled sulfur particles size. Separately, 100 mg of carbon sample (AC−H) was dispersed in 100 mL of H_2_O and sonicated for 1 h. Subsequently, the two solutions were mixed together and heated at 70 °C with the aid of a silicon oil bath, and 15 mL of HCl (12 m) was slowly added under vigorous magnetic stirring to achieve the following reaction [Eq. 2]:

(2) $$\mathrm{Na}{}_{2}S{}_{2}O{}_{3}\;\cdot \;5H{}_{2}O\left(\mathrm{aq}\right)+2\mathrm{HCl}\left(\mathrm{aq}\right)\to 2\mathrm{NaCl}\left(\mathrm{aq}\right)+\mathrm{SO}{}_{2}\left(g\right)+S\left(s\right)+6H{}_{2}O$$

After 15 min, the silicon oil bath was removed and the mixture was left under mild magnetic stirring at room temperature for 24 h. Finally, the obtained sulfur‐carbon composite was washed repeatedly with H_2_O, ethanol, and acetone via centrifugation to remove HCl and Triton X‐100, and then dried at 50 °C in an oven overnight. The final sample is indicated as AC‐H@S.

### Sulfur‐carbon powder characterization

The structural features of the AC‐H@S powder were investigated through XRD and Raman spectroscopy. XRD pattern was obtained by a Bruker D8 Discover X‐ray diffractometer exploiting monochromatic CuK_α_ radiation to scan the 2*θ* range between 10 and 80° by using a step size of 0.04° and a rate of 1.05 s step^−1^. Raman spectroscopy was carried out under ambient conditions by using a Renishaw inVia Microscope equipped with a Renishaw CCD Camera (578×400) detector and a 532 nm edge in line focus mode laser. The sulfur content of AC‐H@S was determined by TGA performed through a Mettler Toledo TGA/DSC‐1 from 30 to 800 °C with a heating rate of 10 °C min^−1^ under N_2_ flow. Sample morphology was studied by SEM and TEM using a JEOL JSM‐7800F and a JEOL 2010 electron microscope operating at 200 kV equipped with an Orius Gatan CCD camera, respectively. The elements distribution of the AC‐H@S composite was evaluated via EDS, which was performed on the TEM images through a X‐ACT Cambridge Instrument analyzer.

### Synthesis of the SiO_x_−C material

The synthesis of the SiO_*x*_−C composite was achieved through sol‐gel method, as reported elsewhere.^[59]^ 18 g of resorcinol was mixed with 58.5 g of formaldehyde at room temperature until a homogeneous mixture was obtained. Subsequently, 21 g of tetraethyl orthosilicate (TEOS) was added to the solution, which was then heated at 70 °C. The dropwise addition of 2 mL of HCl (1 m) to the heated solution catalyzed the formation of a semitrasparent pink gel, which was aged for 24 h at room temperature and then cut into pieces, washed with ethanol to remove residual HCl, and finally annealed at 1000 °C for 10 h under Ar−H_2_ (5 %) flow. The obtained black powder was ground in a mortar.

### Electrode preparation and electrochemical characterization

The electrodes slurries were prepared by dispersing 80 wt% of the active material, either AC‐H@S or SiO_*x*_−C, 10 wt% of Super P carbon (Timcal) as conductive agent, and 10 wt% of PVDF (Solef® 6020 PVDF) as polymer binder, in 1‐methyl‐2‐pyrrolidone (NMP, Sigma‐Aldrich). The slurries containing AC‐H@S and SiO_*x*_−C were coated on a carbon cloth foil (GDL, ELAT LT1400 W, MTI Corp.) and a Cu foil (MTI Corp.), respectively, by using a doctor blade (MTI Corp.). The GDL carbon cloth was used for the sulfur electrode to achieve optimal performances due to its better textural properties compared to common Al.[^82^, ^83^] Then, the electrode films were heated at 50 °C for 5 h under air and subsequently cut into disks of 14 mm diameter, which were dried at 45 °C overnight under vacuum to remove residual traces of water and NMP. The active material loading on the final electrodes was of about 1.3 mg cm^−2^ for AC‐H@S and 5.3 mg cm^−2^ for SiO_*x*_−C.

The electrochemical processes of the AC‐H@S and SiO_*x*_−C composites were analyzed in 2032 coin‐type cells (MTI Corp.) assembled in an Ar‐filled glovebox (MBraun, O_2_ and H_2_O content below 1 ppm) by stacking either an AC‐H@S or a SiO_*x*_−C disk as the positive electrode, a 16 mm diameter Celgard foil soaked with the electrolyte as the separator, and a 14 mm diameter lithium metal disk as the negative electrode. The electrolyte solution exploited in this work was obtained by solvating LiTFSI [LiN(SO_2_)_2_(CF_3_)_2_, 99.95 % trace metals basis, Sigma‐Aldrich] conductive salt and lithium nitrate (LiNO_3_, 99.99 % trace metals basis, Sigma‐Aldrich) sacrificial agent in a solution of DOL (C_3_H_6_O_2_, anhydrous, contains ≈75 ppm butylated hydroxytoluene as inhibitor, 99.8 %, Sigma‐Aldrich) and DME (CH_3_OCH_2_CH_2_OCH_3_, anhydrous, 99.5 %, inhibitor‐free, Sigma‐Aldrich) mixed in a 1 : 1 *w*/*w* ratio. LiTFSI and LiNO_3_ were added to the DOL/DME (1 : 1 *w*/*w*) solution to obtain a final concentration of 1 mol kg^−1^ for each salt, as referred to the solvent mass. Prior to electrolyte preparation, LiTFSI and LiNO_3_ were dried under vacuum to 110 and 80 °C, respectively, for 3 days to remove any trace of water, while DOL and DME were dried with the aid of molecular sieves (3 Å, rod, size 1/16 in., Honeywell Fluka) until a water content below 10 ppm was obtained, as measured by a Karl Fischer 899 Coulometer (Metrohm).

CV and EIS tests were performed on a Li/DOL/DME (1 : 1 *w*/*w*), 1 mol kg^−1^ LiTFSI, 1 mol kg^−1^ LiNO_3_/AC‐H@S cell through a VersaSTAT MC Princeton Applied Research (PAR) analyzer. CV measurements were carried out in the 1.8–2.8 V vs. Li^+^/Li potential range by using a scan rate of 0.05 mV s^−1^, while EIS measurements were performed at the OCV condition and after 1, 5, and 10 CV cycles in the 500 kHz to 100 mHz frequency range by using a 10 mV alternate voltage signal amplitude. The recorded impedance spectra were analyzed by NLLS method through a Boukamp tool and only fitting with a *χ* ^2^ value of the order of 10^−4^ or lower were considered suitable.[^73^, ^74^]

Galvanostatic cycling tests were performed by using a MACCOR series 4000 battery test system. The electrochemical performances of Li/DOL/DME (1 : 1 *w*/*w*), 1 mol kg^−1^ LiTFSI, 1 mol kg^−1^ LiNO_3_/AC‐H@S half‐cells were evaluated through prolonged discharge/charge cycling at the constant current rate of C/3, and through rate capability measurements employing current values of C/10, C/8, C/5, C/3, C/2, 1 C, and 2 C by increasing the current rate every 5 cycles and lowering it to the initial value of C/10 after 35 cycles. The 1.9–2.8 V voltage range was employed from C/10 to C/2 rates, while tests at 1 C and 2 C were carried out between 1.8 and 2.8 V. Both specific current and specific capacity were referred to the sulfur mass.

The lithium‐ion‐sulfur cells were assembled by coupling the AC‐H@S electrode as cathode with a pre‐lithiated SiO_*x*_−C anode (Li_*y*_SiO_*x*_−C) in 2032 coin‐type cells (MTI Corp.) using the Li_*y*_SiO_*x*_−C/DOL/DME (1 : 1 *w*/*w*), 1 mol kg^−1^ LiTFSI, 1 mol kg^−1^ LiNO_3_/AC‐H@S configuration. SiO_*x*_−C electrodes were pre‐activated through 30 discharge‐charge cycles by employing a constant current rate of 50 mA g^−1^ in the 0.01–2.0 V voltage range in Li/DOL/DME (1 : 1 *w*/*w*), 1 mol kg^−1^ LiTFSI, 1 mol kg^−1^ LiNO_3_/SiO_*x*_−C cells. The Li_*y*_SiO_*x*_−C electrodes were recovered from the above cell disassembled at 0.01 V, washed by using DME, and dried under vacuum for 30 min. Galvanostatic cycling tests were performed on the Li_*y*_SiO_*x*_−C/DOL/DME (1 : 1 *w*/*w*), 1 mol kg^−1^ LiTFSI, 1 mol kg^−1^ LiNO_3_/AC‐H@S full‐cells within the 0.1–2.8 V voltage window at a current rate of C/5. Both specific current and specific capacity of the full‐cells were referred to sulfur mass.

Furthermore, chemical lithiation of the SiO_*x*_−C electrode was performed to achieve a suitable condition for practical application of the Li_*y*_SiO_*x*_−C material in full‐cell. The above lithiated anode was achieved by direct contacting the SiO_*x*_−C electrode with a lithium foil soaked with the DOL/DME (1 : 1 *w*/*w*), 1 mol kg^−1^ LiTFSI, 1 mol kg^−1^ LiNO_3_ electrolyte under a pressure of 2 kg cm^−2^ for selected time regimes.^[78]^ The electrode was then removed from the lithium foil, washed by DME, dried for 30 min under vacuum, and studied in lithium half‐cell and full‐cell using AC‐H@S cathode. Galvanostatic cycling tests of chemically lithiated Li_*y*_SiO_*x*_−C pre‐activated for 30 min, 1, 2, and 14 h, as well as of a pristine SiO_*x*_−C electrode, were performed in lithium half‐cell using the DOL/DME (1 : 1 *w*/*w*), 1 mol kg^−1^ LiTFSI, 1 mol kg^−1^ LiNO_3_ electrolyte at a current rate of 50 mA g^−1^ in the 0.01–2 V voltage range. Specific capacity and current were referred to the SiO_*x*_−C mass.

A further lithium‐ion sulfur cell with the Li_*y*_SiO_*x*_−C/DOL/DME (1 : 1 *w*/*w*), 1 mol kg^−1^ LiTFSI, 1 mol kg^−1^ LiNO_3_/AC‐H@S configuration was assembled by using a Li_*y*_SiO_*x*_−C anode achieved by the above described chemical pre‐lithiation for 48 h and cycled within the 0.1–2.8 V voltage window at a current rate of C/5. SEM‐EDS and XRD measurements were carried out on the AC‐H@S and SiO_*x*_−C electrodes employed for this cell at the pristine state, and ex‐situ after 20 discharge/charge cycles. The SEM images were collected through a Zeiss EVO 40 microscope equipped with a LaB_6_ thermoionic electron gun and the EDS analyses were performed by a X‐ACT Cambridge Instruments analyzer. The XRD patterns were recorded through a Bruker D8 ADVANCE diffractometer employing a CuK_α_ source by performing scans between 10 and 90° in the 2*θ* range at a rate of 10 s step^−1^ with a step size of 0.02°. Prior to perform SEM‐EDS and XRD analyses the electrodes were washed with DME and dried under vacuum for 30 min.

## Conflict of interest

The authors declare no conflict of interest.

## Supporting information

As a service to our authors and readers, this journal provides supporting information supplied by the authors. Such materials are peer reviewed and may be re‐organized for online delivery, but are not copy‐edited or typeset. Technical support issues arising from supporting information (other than missing files) should be addressed to the authors.

Supporting Information
