# Supplementary material for: A Stable High‐Capacity Lithium‐Ion Battery Using a Biomass‐Derived Sulfur‐Carbon Cathode and Lithiated Silicon Anode
Source: ChemSusChem. 2021 Jul 16;14(16):3333–43. doi: 10.1002/cssc.202101069 (PMC8457143; doi:10.1002/cssc.202101069)
Supplement: Supplementary file 1 — Supporting Information [file CSSC-14-3333-s001.pdf]

ChemSusChem

Supporting Information

A Stable High-Capacity Lithium-Ion Battery Using a Biomass-Derived Sulfur-Carbon Cathode and Lithiated Silicon Anode

Vittorio Marangon[+], Celia Hernández-Rentero[+], Mara Olivares-Marín, Vicente Gómez-Serrano, Álvaro Caballero, Julián Morales,* and Jusef Hassoun*© 2021 The Authors. ChemSusChem published by Wiley-VCH GmbH. This is an open access article under the terms of the Creative Commons Attribution License, which permits use, distribution and reproduction in any medium, provided the original work is properly cited.

A Stable High Capacity Lithium-Ion Battery Using a Biomass-Derived Sulfur-Carbon Cathode and Lithiated Silicon Anode

Vittorio Marangon[a,†], Celia Hernández-Rentero[b,†], Dr. Mara Olivares-Marín[c], Prof. Vicente Gómez-Serrano[d], Prof. Álvaro Caballero[b], Prof. Julián Morales[b]*, Prof. Jusef Hassoun[a,e,f]*

[a] *Department of Chemical, Pharmaceutical and Agricultural Sciences, University of Ferrara, Via Fossato di Mortara 17, Ferrara 44121, Italy*

[b] *Department of Química Inorgánica e Ingeniería Química, Instituto de Química Fina y Nanoquímica, University of Córdoba, 14071 Córdoba, Spain*

[c] *Department of Ingeniería Mecánica, Energética y de los Materiales, University of Extremadura, Centro Universitario de Mérida, 06800 Mérida, Spain*

[d] *Department of Química Inorgánica, Facultad de Ciencias, University of Extremadura, 06006 Badajoz, Spain*

[e] *Graphene Labs, Istituto Italiano di Tecnologia, Via Morego 30 – 16163 Genova, Italy.*

[f] *National Interuniversity Consortium of Materials Science and Technology (INSTM), University of Ferrara Research Unit, University of Ferrara, Via Fossato di Mortara, 17, 44121, Ferrara, Italy.*

[†] Authors equally contributed.

*Corresponding Authors. E-mail addresses: iq1mopaj@uco.es (Julián Morales), jusef.hassoun@unife.it, jusef.hassoun@iit.it (Jusef Hassoun).

Supporting Information

The active material loadings of about 5.3 mg cm^{-2} for SiO_x-C and 1.3 mg cm^{-2} for AC-H@S allow the achievement of a N/P ratio nearby 1 as shown in Figure S1, which reports steady state voltage profiles of the two materials in lithium half-cell (panel a and b, respectively). It is worth mentioning that the different masses lead to almost the same geometrical surface capacity (that is, of about 1.7 mAh cm^{-2}) due to the different specific capacity of the active materials.

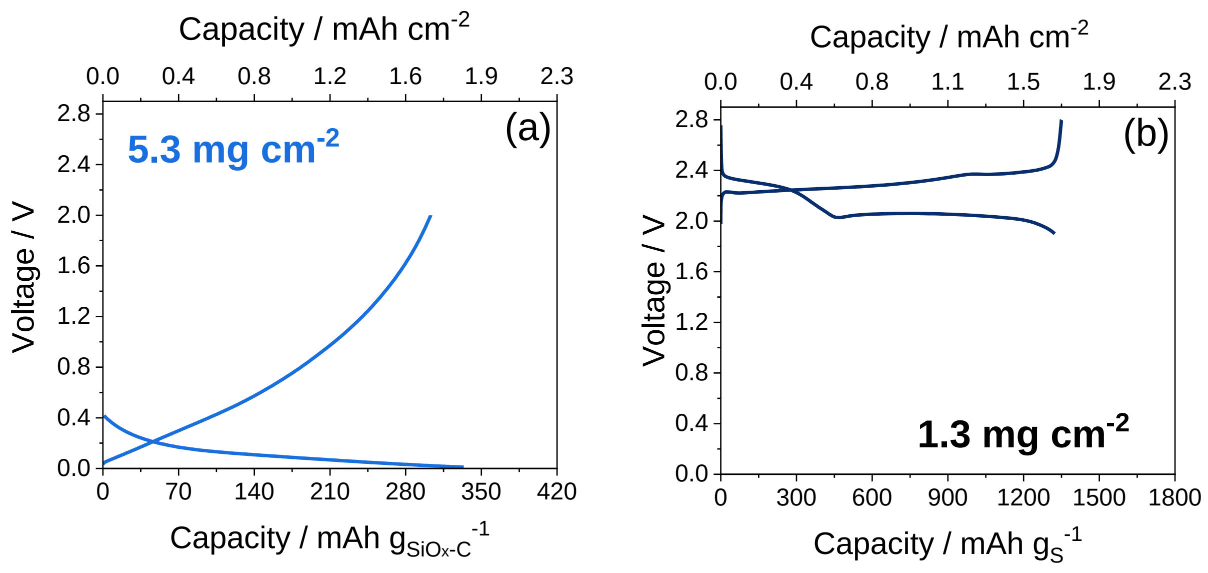

Figure S1. Steady state voltage profile of **(a)** SiO_x-C and **(b)** AC-H@S in lithium half-cell shown to determine the N/P ratio of the full-cell assembled using the two materials and reported in the manuscript (see discussion of Figure 3 and 4). Current values and voltage limits: 50 mA g^{-1}, 0.01 – 2 V for Li/DOL/DME (1:1 w/w), 1 mol kg^{-1} LiTFSI, 1 mol kg^{-1} $LiNO_3$/SiO_x-C cell; and 170 mA g^{-1}, 1.8 – 2.8 V for Li/DOL/DME (1:1 w/w), 1 mol kg^{-1} LiTFSI, 1 mol kg^{-1} $LiNO_3$/AC-H@S cell

A SiO_x-C electrode chemically pre-activated (lithiated) for 48 hours (see Experimental Section) was employed to assemble a Li_ySiO_x-C/AC-H@S full-cell which performed 20 discharge/charge cycles at the constant rate of C/5 (1C = 1675 mA g_S^{-1}), as displayed in Figure 4g of the manuscript. Upon cycling, the electrodes were recovered from the disassembled cell to perform SEM-EDS and XRD analyses on their surfaces. Figure 5 in the Manuscript reports the comparison of SEM images acquired on the surfaces of pristine and cycled AC-H@S and SiO_x-C electrodes, while the corresponding EDS elemental maps are shown in Figure S2 for AC-H@S and Figure S3 for SiO_x-C. The EDS elemental maps of the pristine AC-H@S electrode reveal sulfur aggregates (Fig. S2b) and a relevant presence of phosphorous residues due the activation of the carbon by H_3PO_4 (Fig. S2c). The C (Fig. S2a) and F (Fig. S2d) maps display the uniform distribution of the carbon and the PVDF polymer binder, respectively, used in the electrode slurry (see Experimental Section). It is worth mentioning that the carbonaceous polymeric chain of the PVDF may also contribute to the C signals in Fig. S2a. After cycling, heterogeneously phosphorous-covered particles can be observed (Fig. S2g), while carbon (Fig. S2e), sulfur (Fig. S2f), fluorine (Fig. S2h) and oxygen (inset in Fig. S2h) are uniformly distributed across the electrode surface to form a layer. This layer, which contributes to the SEI film formation upon cycling, is due to electrodeposition of amorphous sulfur upon charge and partial decomposition of the DOL/DME-LiTFSI electrolyte solution.[1] The pristine SiO_x-C electrode exhibits defined domains of the carbon matrices entrapping SiO_x particles, as evidenced by EDS elemental maps of C (Fig. S3a), Si (Fig. S3b) and O (Fig. S3c),[2] the sizes of which decrease upon cycling in full-cell (Fig. S3f) concomitantly to the formation of a uniform layer composed by carbon (Fig. S3e), oxygen (Fig. S3g), fluorine (Fig. S3h) and sulfur (inset Fig. S3h). The contribute of fluorine can also be observed in the pristine SiO_x-C electrode (Fig. S3d) due to the above mentioned PVDF (see Experimental Section).

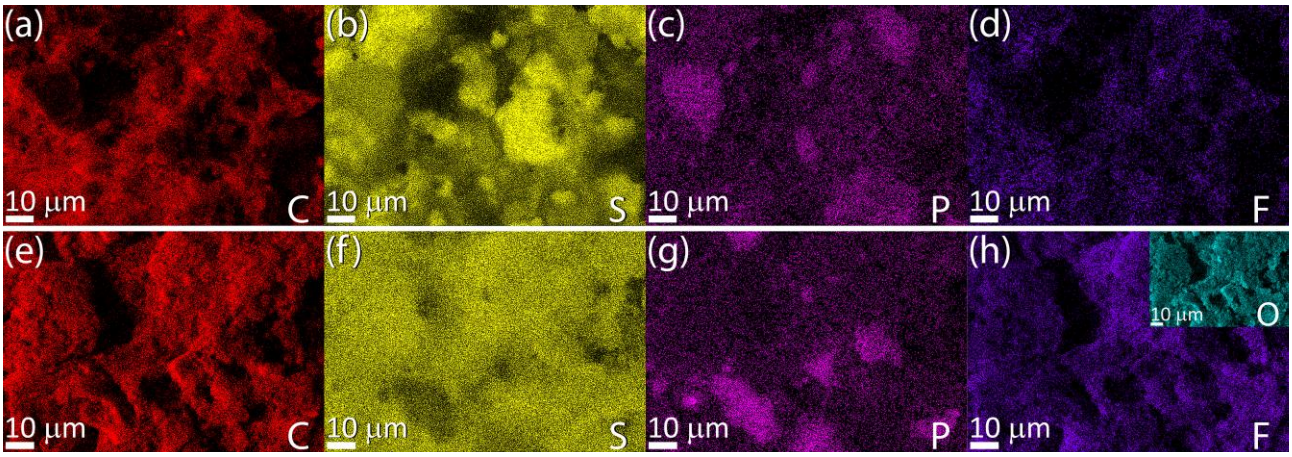

Figure S2. EDS elemental maps recorded on AC-H@S electrodes surface either **(a-d)** at the pristine state or **(e-h)** upon 20 cycles in a Li_ySiO_x-C/DOL/DME (1:1 w/w), 1 mol kg^{-1} LiTFSI, 1 mol kg^{-1} $LiNO_3$/AC-H@S full-cell at the constant current rate of C/5 (1C = 1675 mA g_S^{-1}) in the 0.1 – 2.8 V voltage range (see Fig. 4g in the manuscript for the corresponding voltage profiles).

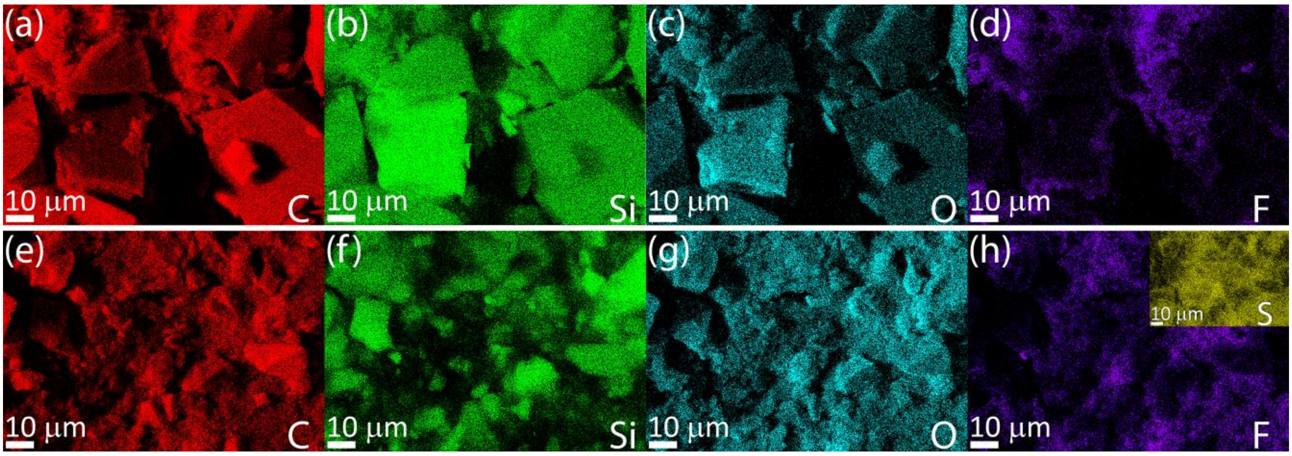

Figure S3. EDS elemental maps recorded on SiO_x-C electrodes surface either **(a-d)** at the pristine state or **(e-h)** upon 20 cycles in a Li_ySiO_x-C/DOL/DME (1:1 w/w), 1 mol kg^{-1} LiTFSI, 1 mol kg^{-1} $LiNO_3$/AC-H@S full-cell at a constant current rate of C/5 (1C = 1675 mA g_S^{-1}) in the 0.1 – 2.8 V voltage range (see Fig. 4g in the manuscript for the corresponding voltage profiles).

References

[1] Q. Liu, A. Cresce, M. Schroeder, K. Xu, D. Mu, B. Wu, L. Shi, F. Wu, *Energy Storage Mater.* **2019**, *17*, 366.

[2] G. A. Elia, J. Hassoun, *ChemElectroChem* **2017**, *4*, 2164.
